# Supplementary material for: Homoeologue expression insights into the basis of growth heterosis at the intersection of ploidy and hybridity in Cyprinidae
Source: Sci Rep. 2016 Jun 6;6:27040. doi: 10.1038/srep27040 (PMC4893626; doi:10.1038/srep27040)
Supplement: Supplementary Information [file srep27040-s1.doc]

**SI Appendix for:**

**Homoeologue expression insights into the basis of growth heterosis at the intersection of ploidy and hybridity in Cyprinidae**

**Li Ren^1*^, Wuhui Li^1*^, Min Tao^1*^, Qinbo Qin^1*^, Jing Luo**^2^**, Jing Chai**^2^**, Chenchen Tang^1^, Jun Xiao^1^, Xiaojun Tang^1^, Guoliang Lin^2^, Chun Zhang^1^, Wei Duan^1^, Yunhan Hong^3^ and Shaojun Liu^1§^**

^1^Key Laboratory of Protein Chemistry and Fish Developmental Biology of Education Ministry of China, College of Life Sciences, Hunan Normal University, Changsha 410081, China

^2^Laboratory for Conservation and Utilization of Bio-resource and Key Laboratory for Animal Genetic Diversity and Evolution of High Education in Yunnan, School of Life Sciences, Yunnan University, Kunming, 650091, Yunnan, China

^3^Department of Biological Sciences, National University of Singapore, Singapore 117543, Singapore

**^§^**Corresponding author:

Professor Shaojun Liu: lsj@hunnu.edu.cn

Key Laboratory of Protein Chemistry & Developmental Biology of State Education Ministry of China, College of Life Sciences, Hunan Normal University

Changsha 410081, China

Tel/Fax: +86-073188873074

^*^These authors contributed equally to this work.

Content

[**SI Table 3**](#__RefHeading___Toc446496185)

[**SI Figures 9**](#__RefHeading___Toc446496186)

# SI Table

Supplementary table S1. The basic information of maternal *Carassius auratus* red var. and paternal *Cyprinus carpio* reference coding sequences in our study

|  | *C. auratus* red var. | *C. carpio* |
| --- | --- | --- |
| Number of annotated genes | 20,169 | 20,235 |
| Number of contig | 39,069 | 52,610 |
| Large contig (≥ 1000bp) | 23,226 | 27,751 |
| Max contig length (bp) | 90,843 | 760,200 |
| Mean contig length (bp) | 1637.7 | 1507.6 |
| N50 length (bp) | 2259 | 2164 |

Supplementary table S2: Summary statistics of the transcriptome data in liver

|  | Number of libraries | Total number of reads | Total number of bases (Gb) | Total number of Q30 bases (Gb) |
| --- | --- | --- | --- | --- |
| *C. auratus* red var. (♀) | Illumina × 3 | 1.92×10^8^ | 17.3 | 10.8 |
| *C. carpio* (♂) | Illumina × 3 | 1.93×10^8^ | 18.5 | 10.5 |
| F_1_ diploid hybrid | Illumina × 3 | 2.07×10^8^ | 19.9 | 11.3 |
| F_18_ allotetraploid | Illumina × 3 | 1.77×10^8^ | 17.0 | 11.7 |

Supplementary table S3. The genes of novel expression or expression silencing pattern between the hybrids with their origin parents in growth-related genes (at threshold of 10 reads homoeologue per million reads)

| Gene symbol | RCC-RPKM | CC-RPKM | F_1_-RPKM | F_18_-RPKM | Ensembl Protein ID | GO Term Accession | GO Term Name |
| --- | --- | --- | --- | --- | --- | --- | --- |
| *il12a* | 10.6 | / | / | 11.1 | ENSDARP00000056760 | GO:0008083 | Growth factor activity |
| *fgf6a* | / | 14.7 | / | / | ENSDARP00000010978 | GO:0008083 | Growth factor activity |
| *fgf12a* | / | / | 15.6 | / | ENSDARP00000036436 | GO:0008083 | Growth factor activity |
| *gdf9* | 13.7 | / | 10.0 | / | ENSDARP00000008475 | GO:0008083 | Growth factor activity |
| *igfbp5a* | / | 12.5 | 15.6 | / | ENSDARP00000057368 | GO:0001558 | Regulation of cell growth |

Supplementary table S4. The basic information of growth-related homoeolgs expression in two hybrids and their origin parents

| Gene symbol | Reference sequences (RCC) | Reference sequences (CC) | Ensembl protein ID | RCC | CC | F_1_-R-HM | F_1_-C-HM | F_18_-R-HM | F_18_-C-HM |
| --- | --- | --- | --- | --- | --- | --- | --- | --- | --- |
| *zgpat* | CauG_12192 | cycg050299 | ENSDARP00000109806 | 3.4 | 1.9 | 1.2 | 0.7 | 1.4 | 1.7 |
| *wnt5b* | CauG_15858 | cycg003085 | ENSDARP00000041851 | 1.3 | 3.9 | 1.9 | 1.4 | 0.3 | 0.1 |
| *spry2-a* | CauG_32727 | cycg000084 | ENSDARP00000052422 | 13.4 | 23.6 | 11.2 | 17.1 | 2.5 | 3.6 |
| *spry2-b* | CauG_36276 | cycg029915 | ENSDARP00000052422 | 3.9 | 9.7 | 5.1 | 12.1 | 2.9 | 4.0 |
| *smurf1-a* | CauG_32922 | cycg001877 | ENSDARP00000111954 | 3.3 | 1.3 | 1.0 | 0.5 | 0.5 | 0.4 |
| *smurf1-b* | CauG_35401 | cycg001905 | ENSDARP00000111954 | 3.2 | 0.9 | 1.1 | 0.4 | 0.4 | 0.2 |
| *smad7-a* | CauG_01475 | cycg020920 | ENSDARP00000024277 | 17.9 | 16.3 | 43.9 | 107.0 | 5.0 | 2.8 |
| *smad7-b* | CauG_03913 | cycg020919 | ENSDARP00000024277 | 23.8 | 20.0 | 29.8 | 21.1 | 3.9 | 3.0 |
| *smad3b* | CauG_37745 | cycg017035 | ENSDARP00000043454 | 8.5 | 3.4 | 1.6 | 0.8 | 3.8 | 1.5 |
| *psen2* | CauG_21931 | cycg000232 | ENSDARP00000011791 | 8.5 | 4.7 | 3.1 | 2.7 | 7.7 | 2.8 |
| *pdgfb* | CauG_03008 | cycg011397 | ENSDARP00000108907 | 3.2 | 2.0 | 4.9 | 2.2 | 1.6 | 0.7 |
| *pdgfaa* | CauG_10914 | cycg021178 | ENSDARP00000095859 | 1.7 | 1.5 | 3.4 | 0.4 | 0.6 | 0.8 |
| *nrp1a* | CauG_34317 | cycg041727 | ENSDARP00000105696 | 1.5 | 1.0 | 0.3 | 0.7 | 0.5 | 1.1 |
| *mul1a* | CauG_33782 | cycg049939 | ENSDARP00000018598 | 3.3 | 5.9 | 3.7 | 2.3 | 2.3 | 2.5 |
| *insrb* | CauG_25790 | cycg021401 | ENSDARP00000096601 | 2.4 | 2.8 | 1.6 | 3.1 | 0.5 | 0.3 |
| *igfbp2a* | CauG_06516 | cycg007417 | ENSDARP00000068816 | 68.8 | 19.8 | 111.5 | 1.6 | 17.2 | 2.0 |
| *igfbp1b* | CauG_08348 | cycg049174 | ENSDARP00000056452 | 462.0 | 1992.2 | 11327.8 | 9539.2 | 230.2 | 115.2 |
| *igfbp1a-a* | CauG_02172 | cycg014709 | ENSDARP00000019674 | 1087.0 | 164.5 | 10005.2 | 859.5 | 225.6 | 13.8 |
| *igfbp1a-b* | CauG_07367 | cycg007542 | ENSDARP00000019674 | 209.6 | 91.2 | 5794.8 | 273.2 | 178.3 | 30.6 |
| *igf2b* | CauG_22953 | cycg010151 | ENSDARP00000047568 | 76.3 | 21.7 | 76.5 | 22.2 | 10.7 | 52.3 |
| *igf2a-a* | CauG_02335 | cycg037273 | ENSDARP00000016954 | 40.3 | 4.4 | 29.7 | 3.8 | 17.8 | 32.1 |
| *igf2a-b* | CauG_27872 | cycg036116 | ENSDARP00000016954 | 158.4 | 4.7 | 13.7 | 8.2 | 14.9 | 36.2 |
| *ids* | CauG_16122 | cycg016470 | ENSDARP00000096983 | 3.8 | 27.1 | 0.7 | 3.2 | 1.1 | 2.8 |
| *gpc4* | CauG_10387 | cycg012804 | ENSDARP00000007058 | 19.7 | 37.7 | 3.5 | 3.8 | 2.7 | 2.3 |
| *gdf2* | CauG_07067 | cycg037054 | ENSDARP00000076657 | 3.8 | 1.4 | 0.4 | 0.3 | 0.4 | 0.5 |
| *fgf23* | CauG_38649 | cycg023915 | ENSDARP00000067387 | 36.1 | 17.5 | 3.3 | 0.8 | 4.3 | 2.4 |
| *ext2* | CauG_11410 | cycg046379 | ENSDARP00000073621 | 3.5 | 1.4 | 0.7 | 0.7 | 0.9 | 1.0 |
| *esm1* | CauG_08287 | cycg021031 | ENSDARP00000078980 | 57.0 | 23.3 | 133.0 | 138.5 | 61.3 | 36.3 |
| *dusp22b* | CauG_21565 | cycg001522 | ENSDARP00000058288 | 44.0 | 28.7 | 21.3 | 19.1 | 53.6 | 19.9 |
| *dll4* | CauG_21407 | cycg032782 | ENSDARP00000094097 | 2.0 | 3.5 | 2.2 | 3.9 | 1.5 | 2.0 |
| *diexf* | CauG_09108 | cycg042380 | ENSDARP00000011386 | 1.3 | 1.0 | 0.8 | 0.5 | 0.6 | 0.6 |
| *ctnnb1* | CauG_05763 | cycg002126 | ENSDARP00000032935 | 10.6 | 4.2 | 1.7 | 0.6 | 3.4 | 2.1 |
| *bmp7a* | CauG_25010 | cycg030806 | ENSDARP00000115518 | 13.1 | 0.9 | 1.2 | 3.1 | 1.6 | 2.9 |
| *bmp2b* | CauG_13025 | cycg019834 | ENSDARP00000107417 | 1.2 | 10.5 | 1.4 | 3.4 | 0.1 | 2.0 |

Abbreviation: HM, homoelogs.

Supplementary table S5. Universal primers that were used to clone the *vasa* genes in hybrids and their origin parents.

|  | Forward primers (+) | Reverse primers (-) | Accession No. |
| --- | --- | --- | --- |
| *vasa* | AGGAAACGGGAAGGCAAACA | ATGTTCCCACAGCGTCCGG | KP661177 (R), KP661178 (C) |

Supplementary table S6. Statistical data of R and C homoeologous sequences

| Gene symbol | NO. SNPs in homoeologue-primers (R, C) |
| --- | --- |
| *igf1* | 10, 10 |
| *ghr* | 7, 7 |
| *igf2* | 7, 9 |
| *tab1* | 6, 6 |
| *bmp4* | 9, 9 |
| *vasa* | 6, 6 |
| *mstn* | 5, 5 |

Supplementary table S7. Primers that were used to determine the relative expression level of homoeologous genes in hybrids and their parents by real-time PCR.

|  | Forward primers (+) | Reverse primers (-) |
| --- | --- | --- |
| *igf1*-R-HM | CGTAAAGCCCGGCAAAACT | TAGGAAGAGTGGCTTTGTCCAG |
| *igf1*-C-HM | TGTAAAGCCTGGAAAAAGT | CAGGCAGAGTGGCTATTTCCAG |
| *ghr*-R-HM | GTCTTCTACATGACAAAACATGCC | GGAGGTCCAGATCTGTGTGAAA |
| *ghr*-C-HM | TCTTCTACCAGACAAAAAATTTCCT | CGAGGTCCAGATCTGTGTGAAG |
| *igf2*-R-HM | CCAGGAGGGCCTCTTGATCTG | ACGATGGCAGGTTGTCTTTC |
| *igf2*-C-HM | GTGCAAAACCCATGAAGTCTGA | CAAGCAGGATGGATGGTACTCC |
| *tab1*-R-HM | AGCATCACACTGCAGCAGACAT | GTGAGTGAGCCGTCTGCGAGT |
| *tab1*-C-HM | CTGCATCACGACGCATACGTG | AGGCATCACCAGAGACAGCATC |
| *bmp4*-R-HM | CGACGTGAGTCCGGCCGT | CGGGTGACTTTTTCCGTCGTGT |
| *bmp4*-C-HM | TTTGACGTGAGCTCAGCCGT | TGGGTGACTTTTGCCGTCATG |
| *vasa*-R-HM | GCTGCTTGATCTGCTCAGGTC | GCTGCTTGATCTGCTCAGGTC |
| *vasa*-C-HM | GCTGCTTGAACTACTCAGATC | AGACATCTTCTCTTGACAGA |
| *mstn*-R-HM | GCGGAAGAAACGACCACTGTT | ACGTCACTGCTGCGTTCACG |
| *mstn*-C-HM | GCGGAAGAAGCAACCACCG | GACGTCACTCCTGCGTTCACA |

# SI Figures

**
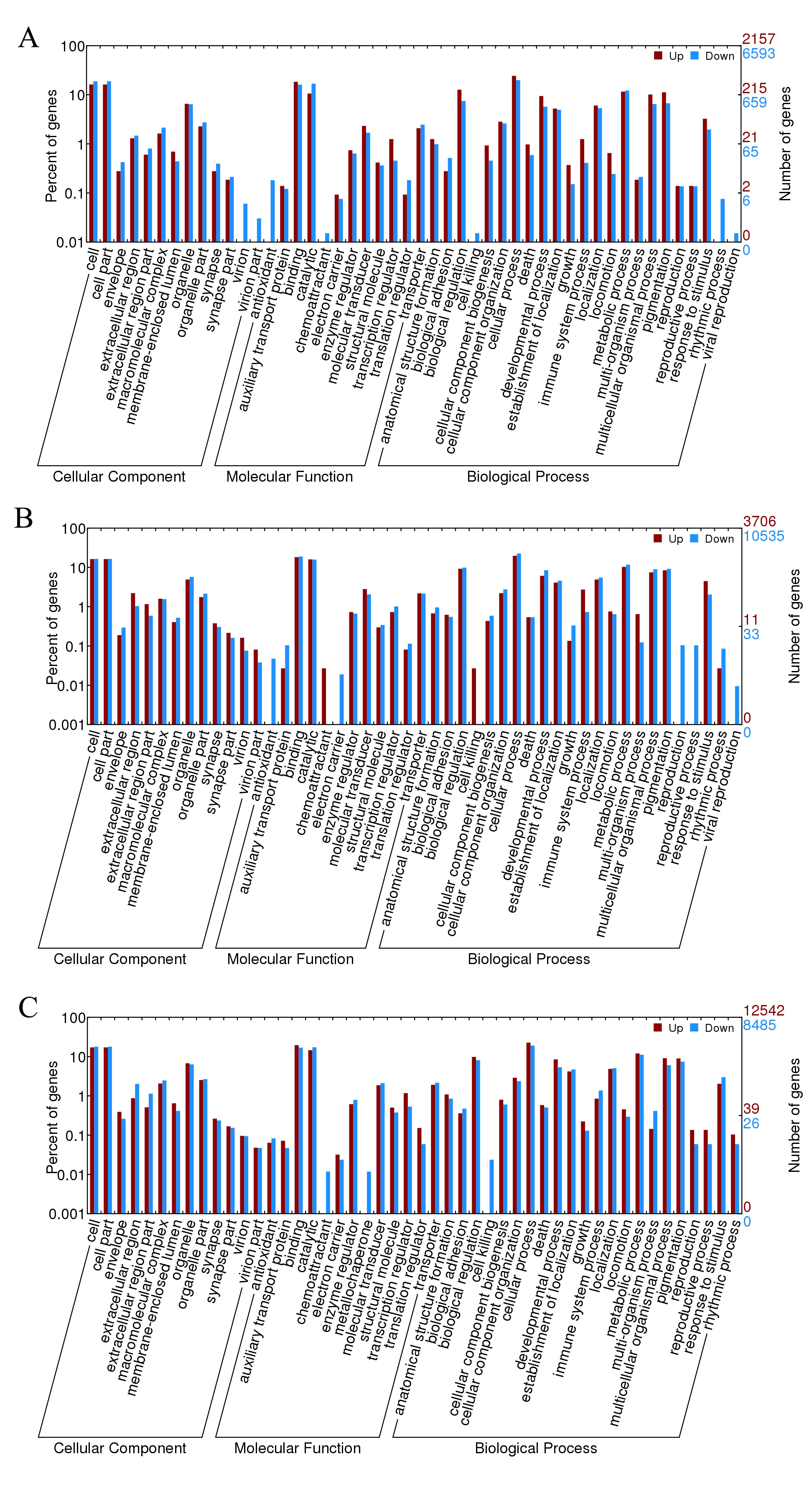
**

**Supplementary Fig. S1.** Gene ontology (GO) assignments for the significantly differential expression genes among two hybrid offspring and MPV. A. GO assignments (level 2) were used to predict the function distribution of up/down- regulated genes between F_1_ and MPV. B. GO assignments (level 2) showed the function distribution of up/down- regulated genes between F_18_ and MPV. C. GO assignments (level 2) were used to predict the function distribution of up/down- regulated genes between F_1_ and F_18_.


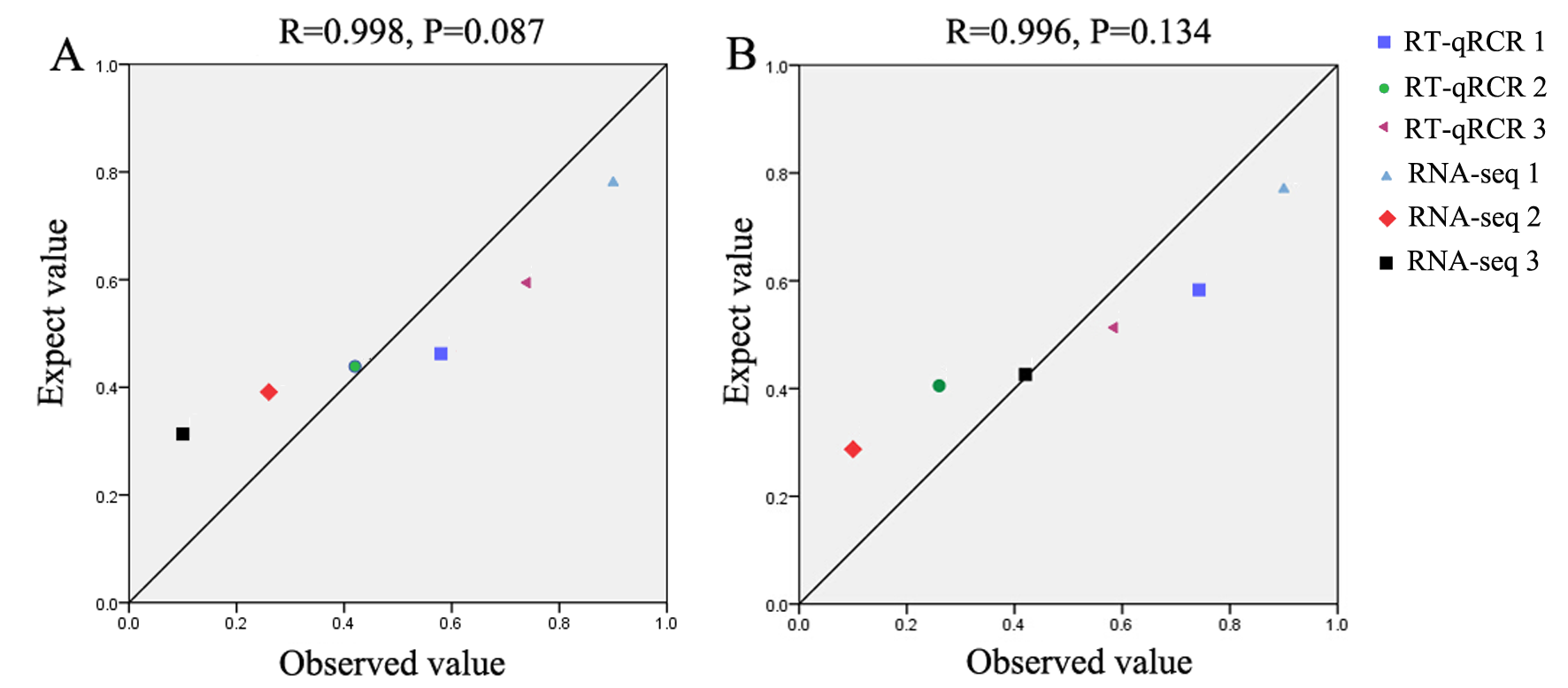


**Supplementary Fig. S2.** Multiple linear regression analysis on homoeologue expression of *bmp4* and *igf2* between the method of RNA-seq and qRT-PCR. A. Homoeologue expression level of *bmp4* gene indicated a positive correlation between RNA-seq and qRT-PCR. B. Homoeologue expression level of *igf2* gene showed a slightly positive correlation between RNA-seq and qRT-PCR.


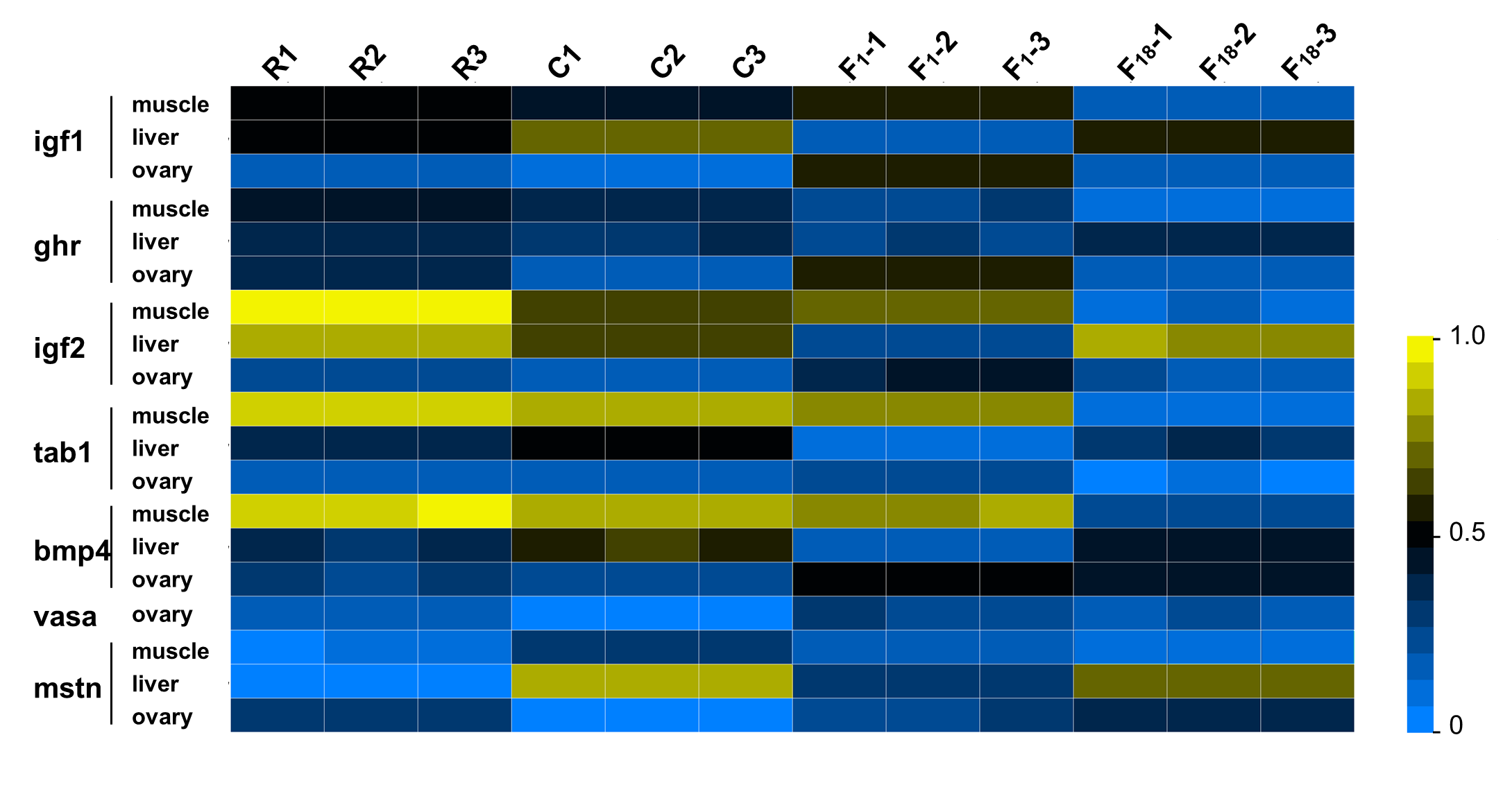


**Supplementary Fig. S3.** Homoeologue expression bias in various organs of two hybrid offspring. A. The maternal expression bias in muscle is estimated by the CT ratio of R homoeologue to C homoeologue in F_18_, and the middle expression pattern is illustrated in the F_1_ individuals. B. The maternal expression bias in liver is estimated by the CT ratio of R homoeologue to C homoeologue in F_1_, and the middle expression pattern is illustrated in the F_18_ individuals. C. The maternal expression bias in ovary is estimated by the CT ratio of R homoeologue to C homoeologue in the two hybrid offspring.


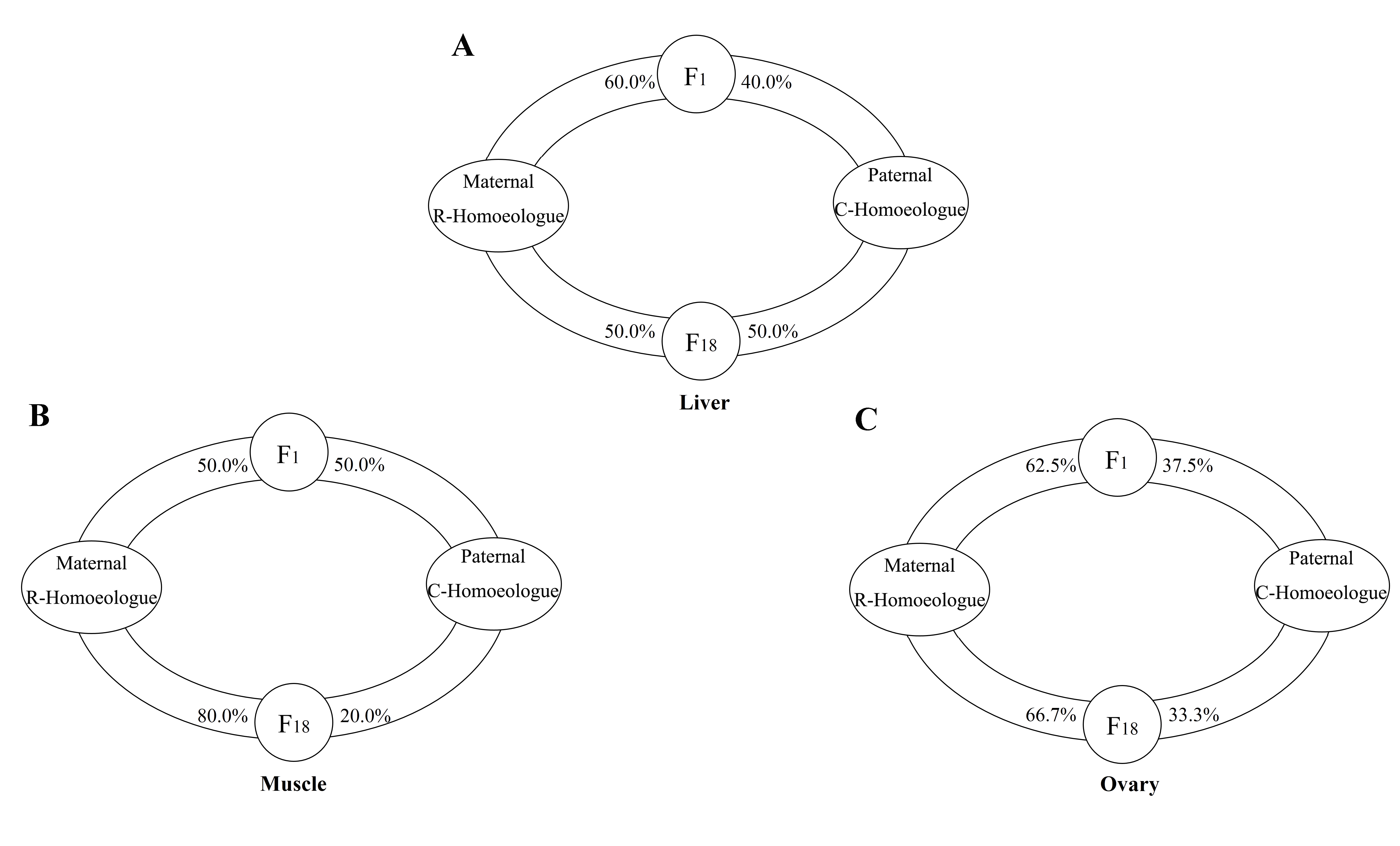


**Supplementary Fig. S4.** Hierarchical clustering analysis of seven genes in various tissues of parents (R1–3 and C1–3) and their three hybrid offspring (F_1_-1–3, and F_18_-1–3) in combination with R and C homoeologue expression. The transcripts with a high expression level are indicated in yellow, and those with a low expression level are indicated in blue.

**
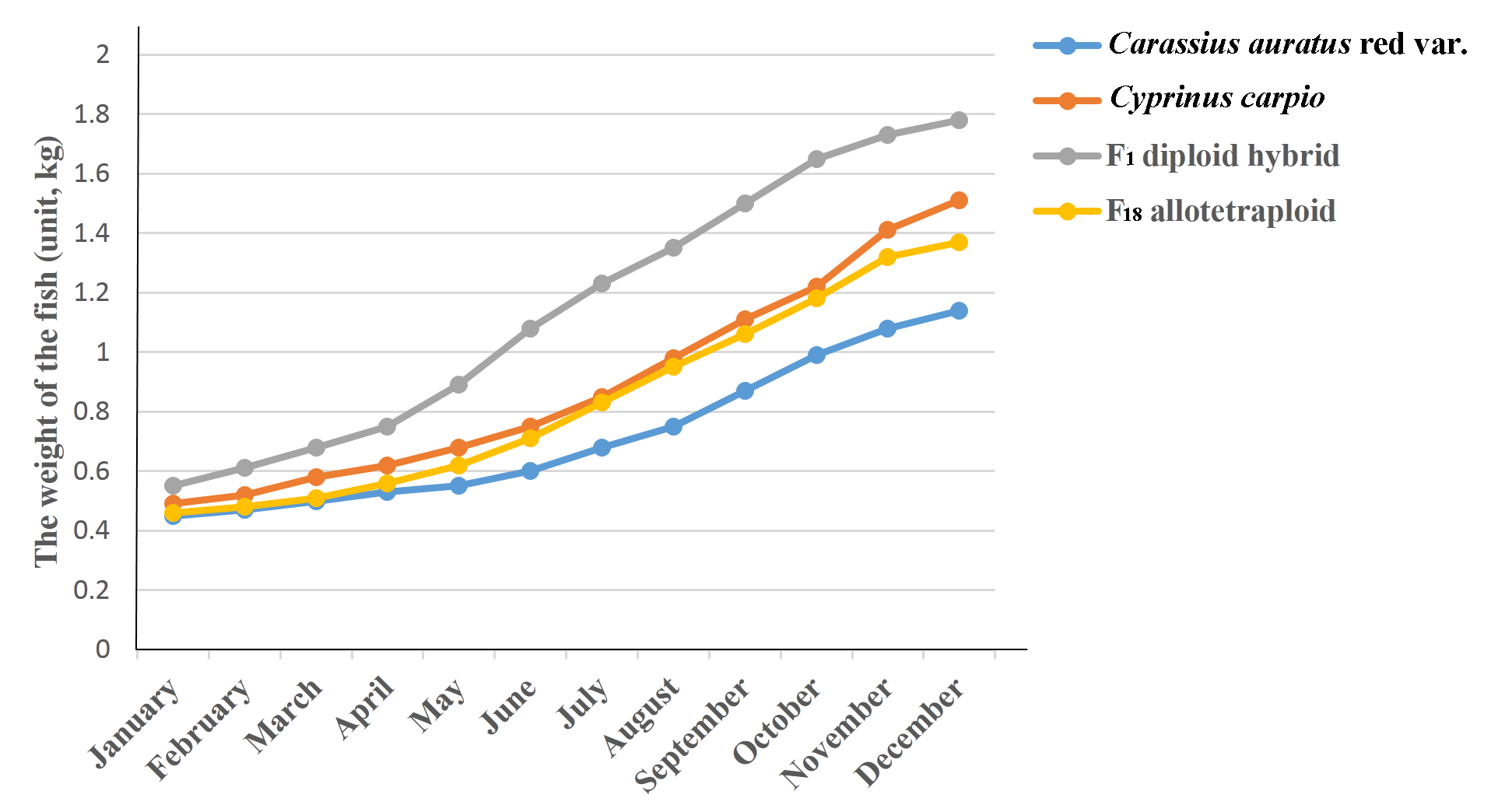
**

**Supplementary Fig. S5.** Comparisons of growth rates of maternal *C. auratus* red var., paternal *C. carpio* and their hybrid progeny (unit, kg).

**
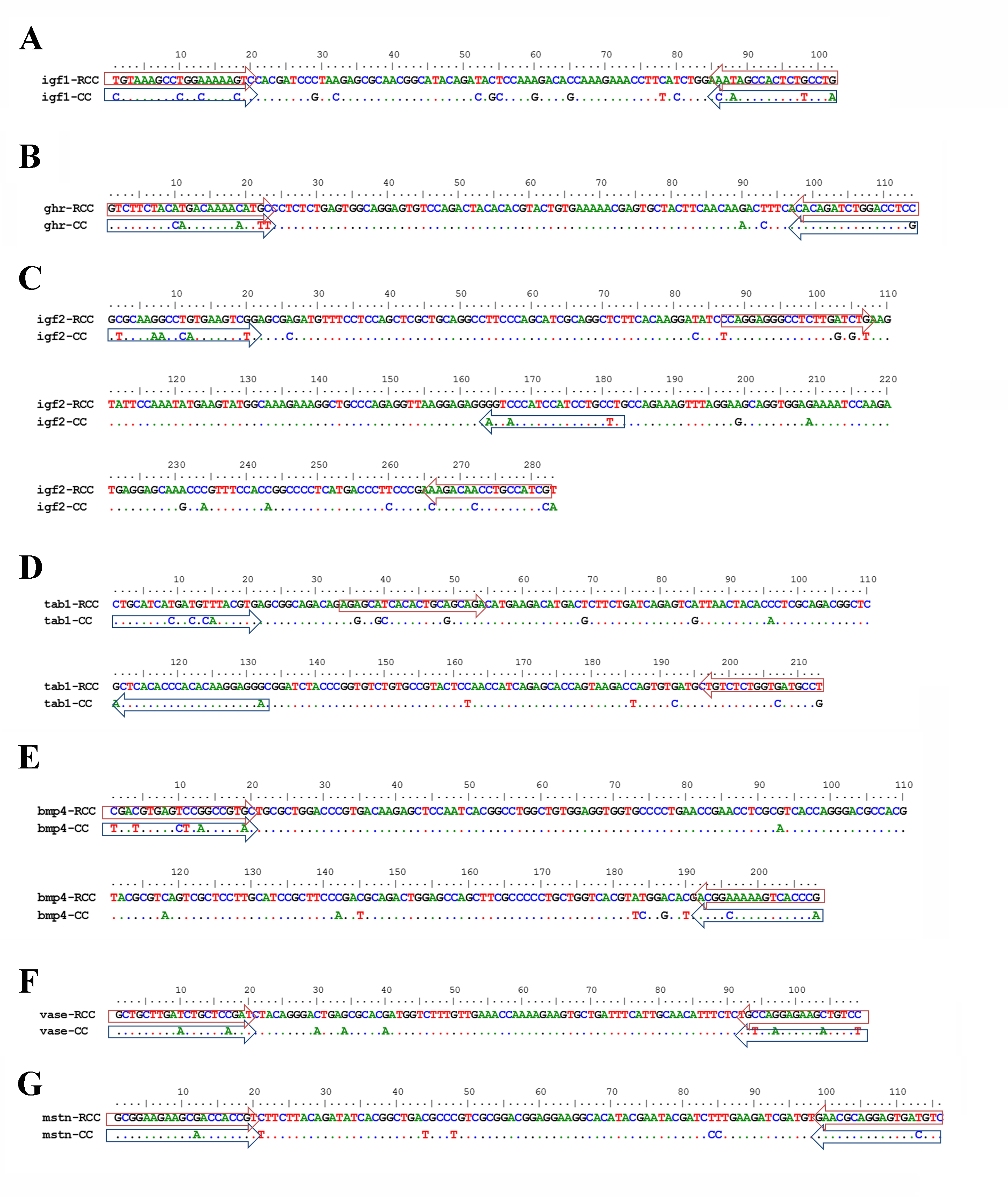
**

**Supplementary Fig. S6.** The alignment of R and C homoeologs and the display of qRT-PCR primers in seven genes. A. The gene of *igf1*. B. The gene of *ghr*. C. The gene of *igf2*. D. The gene of *tab1*. E. The gene of *bmp4*. F. The gene of *vasa*. G. The gene of *mstn*.
